# Ethnicity-Specific Thyroid Hormone Sensitivity Indices and Their Association With Metabolic Syndrome in Euthyroid South Asian Adults

**DOI:** 10.7759/cureus.115062

**Published:** 2026-08-24

**Authors:** Atta Okasha, Ali Haider, Hassan Rizwan, Muhammad Ali Sumbal, Fathima Shasna Thuvan Dharvis, Olumide A Mogbojuri, Hira Shaheen, Tosin Ayantoyinbo, Hammad Jamshaid, Shakil Ahmad, Ume Rubab

**Affiliations:** 1 Healthcare Management with Artificial Intelligence, Arab Academy for Management, Banking and Financial Sciences, Cairo, EGY; 2 Internal Medicine, Nishtar Medical University, Multan, PAK; 3 Medicine, Divisional Headquarters Teaching Hospital, Mirpur, PAK; 4 Emergency Medicine, Nishtar Medical University, Multan, PAK; 5 General Medicine, Aster Hospital Mankhool, Dubai, ARE; 6 General Practice, University of Lagos, Lagos, NGA; 7 General Practice, Fatima Jinnah Medical University, Lahore, PAK; 8 Internal Medicine, BronxCare Health System, New York, USA; 9 Family and Community Medicine, Jalalabad State University, Jalalabad, KGZ; 10 Medicine, Ispahani Islamia Eye Institute and Hospital, Dhaka, BGD; 11 Medicine, Rawalpindi Medical University, Rawalpindi, PAK

**Keywords:** euthyroid adults, metabolic syndrome, thyroid hormone sensitivity, tshi, tt4ri

## Abstract

Background: Metabolic syndrome (MetS) is a combination of interrelated cardiometabolic abnormalities that include abdominal obesity, dyslipidemia, high blood pressure, and insufficient glucose control. Recent studies indicate that sensitivity to altered thyroid hormone, even in euthyroid subjects, can be a contributor to metabolic risk. The present study assessed the relationship between thyroid hormone sensitivity indices, namely the Free Triiodothyronine/Free Thyroxine (FT3/FT4) ratio, the Thyroid-Stimulating Hormone Index (TSHI), the Thyrotropin T4 Resistance Index (TT4RI), and MetS among euthyroid South Asians.

Methods: This cross-sectional observational study included 553 euthyroid adults aged 18 to 60 years, evaluated using demographic, anthropometric, and biochemical measurements. We calculated thyroid hormone sensitivity indices (FT3/FT4 ratio, TSHI, and TT4RI) using standard formulas, and defined prevalent MetS according to the IDF South Asian criteria. We conducted Spearman correlation analyses, group comparisons, ROC analysis, and binary logistic regression.

Results: Metabolic syndrome was present in 115 (20.8%) participants. TSHI and TT4RI were significantly correlated with several metabolic components, including waist circumference, blood pressure, triglycerides, fasting glucose, and BMI, and were negatively correlated with HDL cholesterol (p<0.001). TT4RI was significantly associated with prevalent MetS after adjustment for relevant covariates (OR=1.18, 95% CI: 1.02-1.37, p=0.027). Both indices showed modest discriminatory performance.

Conclusion: MetS was associated with decreased thyroid hormone sensitivity, particularly higher TT4RI levels, in euthyroid South Asian adults. TSHI and TT4RI may serve as complementary markers associated with prevalent metabolic syndrome; however, these findings should be interpreted cautiously and require validation in prospective longitudinal studies.

## Introduction

Metabolic syndrome (MetS) is a complex of interconnected metabolic disorders, such as central obesity, dyslipidemia, high blood pressure, and insulin resistance, that is a major risk factor for cardiovascular diseases (CVDs) and type 2 diabetes mellitus (T2DM) [1,2]. It is becoming increasingly frequent globally, and even among people with lower body mass, the proportion of vulnerable people is disproportionately high due to the interplay of genes, environment, and lifestyle [3,4]. Preventive measures for MetS should be implemented as early as possible by promptly identifying at-risk patients to reduce cardiometabolic complications in the long run [5]. Metabolic syndrome (MetS) affects approximately 25% of adults worldwide and remains a major public health challenge. In Pakistan, the pooled prevalence of MetS among apparently healthy adults has been reported to be 28.8%. Thyroid dysfunction, particularly hypothyroidism, has been associated with metabolic abnormalities; however, the role of impaired thyroid hormone sensitivity in euthyroid individuals remains incompletely understood [6-8].

Thyroid hormones are essential regulators of the body's energy, lipid, and glucose metabolism [9,10]. Although overt thyroid dysfunction is a known cause of metabolic aberrations, emerging evidence suggests that alterations in thyroid hormone sensitivity, even within the euthyroid range, may contribute to metabolic risk [11,12]. To quantify tissue sensitivity to thyroid hormones, new indices have been developed, including the Thyroid Feedback Quantile-based Index (TFQI), Thyrotropin T4 Resistance Index (TT4RI), and TSH Index (TSHI), to provide a finer perspective on thyroid-metabolic interactions [13].

Most studies estimating the index of thyroid hormone sensitivity have been conducted in European or East Asian populations [11,14]. However, their predictive utility for prevalent MetS in South Asians has not been thoroughly studied. Ethnicity-specific metabolic conditions, as well as a higher susceptibility to insulin resistance, are typical of this group [15], and thyroid hormone sensitivity may serve as a marker of MetS, which could be useful for risk stratification and intervention.

Objectives

The main aims of the study were to determine the relationship between thyroid hormone sensitivity indices, including the FT3/FT4 ratio, TSH Index (TSHI), and Thyrotropin T4 Resistance Index (TT4RI), and the presence of metabolic syndrome (MetS) in euthyroid South Asian adults. The study also aimed to determine whether thyroid hormone sensitivity indices were independently associated with prevalent MetS after adjustment for conventional clinical and biochemical factors. Finally, the study examined the relationship between thyroid hormone sensitivity indices and individual MetS components, including central obesity, blood pressure, fasting glucose, triglycerides, and HDL cholesterol.

## Materials and methods

Study framework and setting

It was a cross-sectional observational study conducted at a hospital-based facility to investigate the association between thyroid hormone sensitivity indices and prevalent metabolic syndrome (MetS) among euthyroid South Asian adults. All participants were assessed once, during a single study visit, to ensure clinical, biochemical, and anthropometric parameters were normalised. The study aimed to measure small variations in thyroid hormone sensitivity that may increase metabolic risk, even in patients with normal thyroid activity.

Study population

Table 1 presents the study inclusion and exclusion criteria.

**Table 1:** Inclusion and Exclusion Criteria for the Study Population.

Inclusion Criteria	Exclusion Criteria
Age 18-60 Years	History of Thyroid Disease
Euthyroid Based on TSH, FT3, and FT4	Overt Diabetes Mellitus
South Asian Adults	Cardiovascular Disease
Written Informed Consent	Chronic Kidney Disease
Complete Clinical and Laboratory Data	Other Systemic Illnesses Affecting Thyroid Metabolism
-	Missing Laboratory or Anthropometric Data

Sample size and sampling procedure

The sample size was determined a priori to detect a moderate association (r=0.15) between thyroid hormone sensitivity indices and metabolic syndrome components, with 80% power and a 5% significance level. A minimum of 502 participants was required based on the sample size formula for correlation studies. To account for potential exclusions due to missing laboratory or clinical data, we initially recruited 604 participants; after excluding 51 individuals with incomplete data, we included 553 euthyroid adults in the final analysis. Participants with missing clinical, laboratory, or anthropometric data required to calculate thyroid hormone sensitivity indices or classify metabolic syndrome were excluded from the final analysis (complete-case analysis).

A convenience sampling approach was used. Eligible participants attending the hospital outpatient and general medicine clinics who met the inclusion criteria were invited to participate until the target sample size was exceeded to account for potential exclusions due to missing or incomplete data. The above-described inclusion and exclusion criteria were used to obtain a sample of euthyroid South Asian adults without apparent metabolic or thyroid disease. We obtained written informed consent from all participants before enrollment.

Data collection

Demographics and Lifestyle Factors

We used a structured questionnaire to collect demographic and lifestyle data. We recorded age, sex, and ethnic subgroup. Lifestyle variables included smoking status (never smoker, former smoker, and current smoker) and self-reported physical activity, which was categorized as sedentary, moderately active, or highly active based on participants' responses in the demographic questionnaire. Family history of diabetes and hypertension was also recorded.

Clinical Measurements

Standard protocols were followed for anthropometric measurements. Height and weight were measured with a stadiometer and a scale, respectively, and BMI was calculated as weight (in kilograms) divided by height (in meters) squared. The waist circumference, an indicator of central obesity, was measured at the level of the umbilicus while the person was standing. Blood pressure was measured twice after the participant rested for at least five minutes; the mean of the two measurements was calculated to improve accuracy.

Laboratory Measurements

To determine the biochemical parameters, fasting blood was taken after an 8-12-hour overnight fast. These included TSH, FT3, FT4, fasting glucose, triglycerides (TG), and HDL cholesterol. Serum TSH, FT3, and FT4 concentrations were measured in the hospital's certified clinical laboratory using an automated electrochemiluminescence immunoassay (ECLIA) on the Roche Cobas e411 analyzer (Roche Diagnostics, Mannheim, Germany). Fasting glucose, triglycerides, and HDL cholesterol were measured using standard automated biochemical methods according to the manufacturer's protocols and routine internal quality control procedures. 

Calculation of Thyroid Hormone Sensitivity Indices

For the calculation of TSHI and TT4RI, FT4 values were expressed in pmol/L in accordance with the original published formulas [13]. Three thyroid hormone sensitivity indices were then calculated as follows:

FT3/FT4 ratio: this ratio is calculated as FT3/FT4 and reflects the extent to which inactive thyroxine (T4) is converted into active triiodothyronine (T3), which is the thyroid hormone's activity in tissues.

TSH Index (TSHI): the TSH Index is ln (TSH) + 0.1345 x (FT4), which is the sensitivity to pituitary-thyroid feedback. An increase in TSHI indicates reduced sensitivity to feedback.

Thyrotropin T4 Resistance Index (TT4RI): TSH × FT4, showing a mild thyroid resistance at the point of the hypothalamic-pituitary-thyroid axis.

These indices offer a more sensitive assessment of thyroid activity than standard TSH and FT4 levels, which can be used to examine metabolic risk among euthyroid patients.

Definition of Metabolic Syndrome

MetS was defined according to the IDF 2005 criteria for South Asians. The International Diabetes Federation (IDF) defines MetS according to its criteria for South Asians. Central obesity (waist circumference ≥90 cm in men and ≥80 cm in women) was required, in addition to any two of the following: triglycerides ≥150 mg/dL, reduced HDL cholesterol (<40 mg/dL in men or <50 mg/dL in women), blood pressure ≥130/85 mmHg, or fasting glucose ≥100 mg/dL [16].

Statistical analysis

Descriptive statistics summarized the data. Continuous variables were summarized as mean ± standard deviation (SD) or median (interquartile range (IQR)), depending on data distribution, whereas categorical variables were presented as frequencies (n) and percentages (%). Comparisons between the MetS-present and MetS-absent groups were performed using independent t-tests for normally distributed variables or Mann-Whitney U tests for non-normally distributed variables. We first performed univariable logistic regression to estimate crude odds ratios. We then entered clinically relevant variables into multivariable logistic regression models to estimate adjusted odds ratios. Each thyroid hormone sensitivity index (TSHI, TT4RI, and FT3/FT4 ratio) was examined in a separate adjusted model, with adjustment for age, sex, BMI, smoking status, and physical activity based on clinical relevance. Ethnicity was examined separately in subgroup analyses, while family history was not included as a primary adjustment covariate. Multicollinearity among the independent variables in each regression model was assessed using the variance inflation factor (VIF); VIF values ranged from 1.08 to 1.34, indicating no problematic multicollinearity (Appendix A). The significance level was set at p<0.05.

Ethical considerations

The institute's Institutional Review Board of Nishtar Medical College, Multan, Pakistan, IRB Reference Number 2891NMC, approved this study. The research methods were guided by ethical principles set out in the Declaration of Helsinki. All participants provided written informed consent. All data were stored securely, were conditionally identifiable using identification codes, and could only be accessed by the research team (anonymity was maintained).

## Results

The sample size of this study was 553, as shown in Table 2. Age was relatively evenly distributed: 137 (24.8%) participants were aged 18-29 years, 145 (26.2%) aged 30-39 years, 142 (25.7%) aged 40-49 years, and 129 (23.3%) aged 50-60 years. There were 302 (54.6%) males and 251 (45.4%) females. Ethnic distribution included 128 (23.1%) Punjab participants, 122 (22.1%) Bengali participants, 112 (20.3%) Gilgiti participants, 99 (17.9%) Sindhi participants, and 92 (16.6%) Pakhtoon participants. Regarding smoking status, 351 (63.5%) were never smokers, 137 (24.8%) were ex-smokers, and 65 (11.8%) were current smokers. According to the physical activity assessment, 269 (48.6%) were sedentary, 138 (25.0%) were moderately active, and 146 (26.4%) were active. Many participants reported a family history of diabetes and/or hypertension, as follows: 213 (38.5%) had no family history; 133 (24.1%) had diabetes only; 115 (20.8%) had hypertension only; and 92 (16.6%) had both diabetes and hypertension. In addition, 261 (47.2%) had central obesity, 255 (46.1%) had low HDL cholesterol, 132 (23.9%) had elevated triglycerides, 138 (25.0%) had elevated blood pressure, and 114 (20.6%) had elevated fasting glucose. Overall, metabolic syndrome was present in 115 (20.8%) participants and absent in 438 (79.2%) participants, indicating a moderate burden of cardiometabolic risk factors in the study population.

**Table 2:** Frequency Distribution of Study Sample Characteristics (N=553). Note. f:frequency; %: percentage. Values represent counts and valid percentages. N=553 (after exclusion of 51 cases with data quality issues). MetS: metabolic syndrome; IDF:  International Diabetes Federation; DM: diabetes mellitus; HTN: hypertension; IPAQ: International Physical Activity Questionnaire.

Variable	f (N)	%
Age Group	–	–
18–29 years	137	24.8
30–39 years	145	26.2
40–49 years	142	25.7
50–60 years	129	23.3
Gender	–	–
Male	302	54.6
Female	251	45.4
Ethnic Subgroups	–	–
Punjabi	128	23.1
Sindhi	99	17.9
Pakhtoon	92	16.6
Gilgiti	112	20.3
Bengali	122	22.1
Smoking Status	–	–
Never Smoker	351	63.5
Ex-Smoker	137	24.8
Current Smoker	65	11.8
Physical Activity Level (IPAQ-Short)	–	–
Sedentary	269	48.6
Moderate	138	25.0
Active	146	26.4
Family History of DM/HTN	–	–
None	213	38.5
Diabetes Only	133	24.1
Hypertension Only	115	20.8
Both	92	16.6
Central Obesity (IDF South Asian Cutoff)	–	–
Yes	261	47.2
No	292	52.8
Elevated Triglycerides (≥150 mg/dL)	–	–
Yes	132	23.9
No	421	76.1
Low HDL Cholesterol	–	–
Yes	255	46.1
No	298	53.9
Elevated Blood Pressure (≥130/85 mmHg)	–	–
Yes	138	25.0
No	415	75.0
Elevated Fasting Glucose (≥100 mg/dL)	–	–
Yes	114	20.6
No	439	79.4
Metabolic Syndrome	–	–
Present	115	20.8
Absent	438	79.2

Table 3 demonstrates that TSHI showed positive associations with waist circumference (ρ=0.180, p<0.001), systolic blood pressure (ρ=0.155, p<0.001), diastolic blood pressure (ρ=0.162, p<0.001), triglycerides (ρ=0.175, p<0.001), fasting blood glucose (ρ=0.160, p<0.001), and BMI (ρ=0.205, p<0.001), while showing a negative association with HDL cholesterol (ρ=−0.171, p<0.001). Similarly, TT4RI was positively correlated with waist circumference (ρ=0.201, p<0.001), systolic blood pressure (ρ=0.168, p<0.001), diastolic blood pressure (ρ=0.177, p<0.001), triglycerides (ρ=0.188, p<0.001), fasting blood glucose (ρ=0.181, p<0.001), and BMI (ρ=0.214, p< 0.001), and negatively correlated with HDL cholesterol (ρ=−0.186, p<0.001). In contrast, no significant associations were observed between the FT3/FT4 ratio and the metabolic syndrome components; the weak correlation with DBP (ρ=0.084) did not remain significant after Bonferroni correction.

**Table 3:** Spearman Rank Correlations Between Thyroid Sensitivity Indices and Metabolic Syndrome Components (N=553) Values are Spearman correlation coefficients (ρ) with p-values in parentheses. SBP: systolic blood pressure; DBP: diastolic blood pressure; TG: triglycerides; HDL: high-density lipoprotein; FBG: fasting blood glucose; BMI: body mass index. Bonferroni-adjusted α=0.0024; *** p<0.001 considered significant.

Thyroid Sensitivity Index	Waist (cm)	SBP (mmHg)	DBP (mmHg)	TG (mg/dL)	HDL (mg/dL)	FBG (mg/dL)	BMI (kg/m²)
FT3/FT4 Ratio	-0.032	-0.013	0.084	-0.006	-0.022	0.025	-0.038
TSH Index (TSHI)	0.180***	0.155***	0.162***	0.175***	-0.171***	0.160***	0.205***
Thyrotropin T4 Resistance Index (TT4RI)	0.201***	0.168***	0.177***	0.188***	-0.186***	0.181***	0.214***

Table 4 shows that participants with metabolic syndrome had higher TSHI values than those without metabolic syndrome (0.82 ± 0.69 vs 0.67 ± 0.65, p=0.035) and higher TT4RI values (median 2.18 vs 1.87, p=0.008). In contrast, the FT3/FT4 ratio did not differ significantly between the groups (2.69 vs 2.68, p=0.918). As expected, participants with metabolic syndrome had higher BMI (32.6 vs 25.9 kg/m², p<0.001), waist circumference (93.8 vs 81.0 cm, p<0.001), triglycerides (149 vs 110 mg/dL, p<0.001), systolic blood pressure (120 vs 115 mmHg, p=0.005), and fasting glucose (95 vs 86 mg/dL, p<0.001), along with lower HDL cholesterol (38 vs 47 mg/dL, p<0.001). No significant differences were observed in TSH, FT3, or FT4 between the groups.

**Table 4:** Comparison of Thyroid Sensitivity Indices and Metabolic Parameters Between Metabolic Syndrome Groups (N=553). Values are median (IQR) except TSHI (mean±SD). Mann–Whitney U test was used for most variables; independent t-test for TSHI; MetS: metabolic syndrome; IQR: Interquartile Range; TSH: thyroid-stimulating hormone; FT3: free triiodothyronine; FT4: free thyroxine; FT3/FT4 Ratio: ratio of free triiodothyronine to free thyroxine; TT4RI: thyroxine resistance index; BMI: body mass index; BP: blood pressure; †TSHI: thyroid hormone sensitivity index.

Variable	MetS Absent (N=438)	MetS Present (N=115)	Test statistic	p-value	Effect size/95% CI
FT3/FT4 Ratio	2.68 (0.80)	2.69 (0.79)	U=25047	0.918	—
TSHI	0.67±0.65	0.82±0.69	t=2.12	0.035	Mean difference 0.15 (0.01–0.29)
TT4RI	1.87 (1.91)	2.18 (2.08)	U=21483	0.008	—
TSH (mIU/L)	1.72 (1.64)	1.72 (1.62)	U=24262	0.545	—
FT3 (pg/mL)	3.09 (0.67)	3.09 (0.68)	U=24839	0.820	—
FT4 (pmol/L)	14.80 (3.22)	14.82 (3.09)	U=24237	0.534	—
BMI (kg/m²)	25.9 (6.8)	32.6 (5.8)	U=7364	<0.001	—
Waist circumference (cm)	81.0 (14.2)	93.8 (12.6)	U=8023	<0.001	—
Triglycerides (mg/dL)	110 (52)	149 (62)	U=13221	<0.001	—
HDL cholesterol (mg/dL)	47 (12)	38 (11)	U=14618	<0.001	—
Systolic BP (mmHg)	115 (17)	120 (19)	U=20868	0.005	—
Fasting glucose (mg/dL)	86 (17)	95 (18)	U=15315	<0.001	—

Table 5 shows that subgroup differences in thyroid sensitivity indices are statistically significant for both genders and age groups. Part A showed that males had a higher mean rank of TT4RI than females (289.45 vs. 262.03), and the difference was statistically significant (p=0.045), meaning that thyrotropin resistance to T4 is relatively higher in males. TT4RI and TSHI were significantly different across age groups in Part B (p=0.038 and p=0.030, respectively). Mean ranks of the two indices showed a gradual increase with age, with the highest values in the 50-60 years group, indicating an increase in thyroid hormone resistance indices with age. Altogether, these results demonstrate that thyroid sensitivity, as measured by TSHI and TT4RI, varies by gender and age.

**Table 5:** Subgroup Differences in Thyroid Sensitivity Indices by Gender and Age Group (N=553). Note. Gender differences were assessed using the Mann–Whitney U test, and age-group differences were assessed using the Kruskal–Wallis test. Values are presented as mean ranks. Higher mean ranks indicate higher index values. TT4RI: thyrotropin T4 resistance index; TSHI: TSH index; *p<0.05.

Variable	Comparison	Group	Mean Rank	Test Statistic	p-value
TT4RI	Gender	Male (N=302)	289.45	U=34143	0.045*
		Female (N=251)	262.03		
TSHI	Age Group	18–29 (N=137)	249.22	H=8.412	0.038*
		30–39 (N=145)	269.40		
		40–49 (N=142)	288.83		
		50–60 (N=129)	302.02		
TT4RI	Age Group	18–29 (N=137)	248.68	H=8.937	0.030*
		30–39 (N=145)	268.67		
		40–49 (N=142)	289.23		
		50–60 (N=129)	302.97		

Table 6 presents the ROC curve analysis of thyroid sensitivity indices for metabolic syndrome. The TSHI and TT4RI showed statistically significant but limited discriminatory performance, with AUCs of 0.596 (p=0.002) and 0.590 (p=0.004), respectively. In contrast, the FT3/FT4 ratio did not demonstrate significant discriminatory ability for metabolic syndrome (AUC=0.512, p=0.693), indicating performance close to chance level in this cohort.

**Table 6:** Receiver Operating Characteristic (ROC) Curve Analysis of Thyroid Sensitivity Indices for Predicting Metabolic Syndrome. Note. AUC: area under the receiver operating characteristic curve; SE: standard error; CI: confidence interval. Positive state implies that metabolic syndrome is present.

Thyroid Index	AUC	SE	95% CI	p
FT3/FT4 Ratio (Peripheral Conversion Index)	0.512	0.030	0.452–0.572	0.693
TSH Index (TSHI)	0.596	0.031	0.535–0.657	0.002
Thyrotropin T4 Resistance Index (TT4RI)	0.590	0.031	0.529–0.651	0.004

Table 7 presents the results of adjusted binary logistic regression analyses identifying factors associated with metabolic syndrome. After adjustment for potential confounders, TT4RI remained significantly associated with metabolic syndrome (OR=1.18, 95% CI: 1.02-1.37, p=0.027). Increasing age (OR=1.03, p=0.014), female sex (OR=1.94, p=0.013), and higher BMI (OR=1.34, p<0.001) were also independently associated with greater odds of metabolic syndrome. Neither ex-smoking nor current smoking was significantly associated with metabolic syndrome (p=0.744 and p=0.319, respectively). In contrast, active physical activity was associated with lower odds of metabolic syndrome compared with sedentary physical activity (OR=0.34, 95% CI: 0.16-0.70, p=0.004).

**Table 7:** Crude and Adjusted Binary Logistic Regression Models Examining Thyroid Sensitivity Indices and Covariates Associated With Metabolic Syndrome (N=553). B: unstandardized regression coefficient; SE: standard error; OR: odds ratio; CI: confidence interval. Reference categories: male gender, never smoker, and sedentary physical activity. TT4RI: thyrotropin T4 resistance index; BMI: body mass index.

Variable	B	SE	Wald	p value	OR	95% CI
Predictor	Crude Logistic Regression Analysis of Thyroid Sensitivity Indices Predicting Metabolic Syndrome					
TSHI	0.192	0.072	7.11	0.008	1.21	1.05–1.39
TT4RI	0.166	0.078	4.53	0.033	1.18	1.01–1.37
FT3/FT4 Ratio	0.019	0.062	0.09	0.760	1.02	0.90–1.15
Model 1	Adjusted Logistic Regression Model 1: TSHI as Predictor					
TSHI	0.190	0.071	7.16	0.007	1.21	1.05–1.39
Age (Years)	0.029	0.012	5.84	0.014	1.03	1.01–1.05
Female Sex	0.652	0.265	6.05	0.014	1.92	1.14–3.22
BMI	0.292	0.033	78.20	<0.001	1.34	1.26–1.43
Ex-Smoker	-0.102	0.322	0.10	0.752	0.90	0.48–1.68
Current Smoker	0.381	0.388	0.97	0.325	1.46	0.68–3.12
Moderate Physical Activity	0.118	0.305	0.15	0.700	1.13	0.62–2.05
Active Physical Activity	-1.082	0.380	8.11	0.004	0.34	0.16–0.71
Model 2	Adjusted Logistic Regression Model 2: TT4RI as Predictor					
TT4RI	0.166	0.075	4.90	0.027	1.18	1.02–1.37
Age (Years)	0.029	0.012	5.84	0.014	1.03	1.01–1.05
Female Sex	0.660	0.266	6.15	0.013	1.94	1.15–3.25
BMI	0.295	0.033	80.00	<0.001	1.34	1.26–1.43
Ex-Smoker	-0.105	0.322	0.11	0.744	0.90	0.48–1.69
Current Smoker	0.386	0.389	0.99	0.319	1.47	0.69–3.13
Moderate Physical Activity	0.120	0.306	0.15	0.695	1.13	0.62–2.06
Active Physical Activity	-1.090	0.381	8.18	0.004	0.34	0.16–0.70
Model 3	Adjusted Logistic Regression Model 3: FT3/FT4 Ratio as Predictor					
FT3/FT4 Ratio	0.019	0.062	0.10	0.759	1.02	0.90–1.15
Age (Years)	0.029	0.012	5.84	0.014	1.03	1.01–1.05
Female Sex	0.654	0.265	6.08	0.014	1.92	1.14–3.22
BMI	0.293	0.033	79.00	<0.001	1.34	1.26–1.43
Ex-Smoker	-0.101	0.323	0.10	0.754	0.90	0.48–1.68
Current Smoker	0.380	0.389	0.96	0.327	1.46	0.68–3.12
Moderate Physical Activity	0.117	0.306	0.15	0.702	1.12	0.62–2.05
Active Physical Activity	-1.080	0.380	8.08	0.004	0.34	0.16–0.71

## Discussion

The research adopted a cross-sectional design to assess the relationships among indices of thyroid hormone sensitivity, including the FT3/FT4 ratio, the TSHI, and TT4RI, and prevalent MetS in euthyroid South Asian adults. In our analysis, there was no significant correlation between individual components of MetS and FT3/FT4. This is consistent with NHANES data, in which FT3/FT4 had only a moderate influence on overall MetS severity [17]. In contrast, poor metabolic characteristics (higher waist circumference, BMI, blood pressure, triglycerides, and fasting glucose; and lower HDL) were associated with higher TSHI and TT4RI. These results are consistent with a prior study, which reported the same associations between TSHI and TT4RI, and between MetS and its elements, substantiating the role of central thyroid resistance in metabolic dysfunction [18].

In our study, TSHI and TT4RI were significantly elevated in the MetS population, whereas FT3/FT4 and individual thyroid hormones showed no significant differences. These results are consistent with earlier studies, which also found that central thyroid resistance indices are more strongly linked to metabolic risk than peripheral thyroid conversion [19,17]. Furthermore, MetS participants had significantly higher BMI, waist circumference, triglycerides, and blood pressure, and lower HDL than those without MetS. These findings are consistent with previous studies showing that central obesity, hyperglycemia, and low HDL are common in MetS and correlate with poor health outcomes [20].

Sex-based differences were also observed: males had much higher TT4RI than females in our study, indicating slightly higher central thyroid resistance. This supports the findings of other studies that demonstrate that increased TT4RI in men has a stronger correlation with metabolic risk and CKM syndrome than in women [21]. Additionally, both TSHI and TT4RI were statistically significantly elevated with age, indicating that thyroid hormone resistance index values increased with age. Likewise, a previous study reported that thyroid sensitivity indices are significantly associated with metabolic phenotypes, particularly across age groups [22].

Receiver operating characteristic analysis demonstrated that TSHI and TT4RI had statistically significant but only modest discriminatory ability for identifying prevalent MetS, whereas the FT3/FT4 ratio showed no meaningful discriminatory ability. In the multivariable analysis, higher TT4RI was significantly associated with greater odds of prevalent MetS after adjustment for age, sex, BMI, smoking status, and physical activity. Earlier research corroborates this, showing that lower sensitivity to thyroid hormones is associated with a higher prevalence of MetS and that demographic variables such as age and sex may influence this association [18,21,22]. BMI was also the strongest associated classical metabolic risk factor for MetS in our study. BMI increased the odds of MetS by an average of 35% per 1 kg/m^2^ increase. Similarly, a prior hospital-based investigation identified obesity as associated with MetS, with excess adiposity as the primary driver of metabolic risk [23]. The status of smoking, however, was not significantly associated with MetS in our analysis. Conversely, large-scale cohort studies have indicated that current smoking is a risk factor for MetS in all BMI categories, indicating that the correlation between smoking and metabolic risk is different by population and study design [24]. Finally, physically active individuals had much lower odds of metabolic syndrome than sedentary participants. These findings align with earlier studies demonstrating that low physical activity levels were associated with higher MetS prevalence, whereas regular physical activity was associated with lower odds of MetS [25].

Overall, the findings suggest that thyroid hormone sensitivity indices, particularly TT4RI, are associated with prevalent MetS in euthyroid South Asian adults. Although these indices may provide additional information beyond conventional metabolic markers, their modest discriminatory performance and the cross-sectional design of this study warrant cautious interpretation. Further prospective multicentre studies are needed to validate their clinical utility.

Limitations

Consider the limitations of this study when interpreting the findings. First, the cross-sectional design, in which all clinical and biochemical data were collected at a single time point, precludes the assessment of temporal relationships or causal inferences between thyroid hormone sensitivity indices and metabolic syndrome. Prospective longitudinal studies are required to confirm these associations. Second, the study used a single-centre, hospital-based convenience sample, which may have introduced selection bias and limited the generalizability of the findings to the broader South Asian population. Patients attending healthcare facilities may differ from the general population in terms of health awareness, comorbidities, and healthcare-seeking behaviours. Third, lifestyle variables, including smoking status and physical activity, were self-reported and may have been subject to recall bias or misclassification. Furthermore, dietary intake was not objectively assessed. Fourth, although the regression analyses were adjusted for several important confounders, residual confounding from unmeasured factors, such as dietary habits and socioeconomic status, cannot be excluded. In addition, insulin resistance markers, such as the homeostatic model assessment for insulin resistance (HOMA-IR), were not evaluated, which may have provided further insight into metabolic function. Finally, although participants from several South Asian ethnic subgroups were included, the sample size within individual subgroups may have been insufficient to detect ethnic-specific differences in thyroid-metabolic associations. Furthermore, although TT4RI demonstrated statistically significant discriminatory ability, its diagnostic performance was modest and should be interpreted with caution. Future multicentre prospective studies are warranted to validate these findings and determine their clinical applicability.

Future directions

Future research should build on these findings as follows. First, longitudinal cohort studies that include repeated measurements of thyroid hormone sensitivity indices are needed to determine whether these measures can predict the association with metabolic syndrome or cardiometabolic disease in the future. Second, community-based population studies would improve the generalizability of the findings by evaluating whether the associations hold in the general South Asian population. Third, molecular and physiological mechanisms of thyroid hormone resistance that lead to insulin resistance, dyslipidemia, and adiposity may be studied in mechanistic detail, clarifying the biological mechanisms underlying these relationships. Fourth, thyroid hormone sensitivity indices can be included in multivariate cardiometabolic risk discrimination to assess whether they improve risk stratification beyond traditional metabolic indices. Finally, future research must investigate the genetic and environmental factors influencing thyroid hormone sensitivity in South Asian populations, as ethnic differences in metabolic predisposition may influence endocrine-metabolic interactions.

## Conclusions

In conclusion, the present study demonstrated that the central thyroid hormone sensitivity indices, TSHI and TT4RI, were significantly associated with prevalent metabolic syndrome and its components in euthyroid South Asian adults. In contrast, the FT3/FT4 ratio showed no significant association despite normal thyroid hormone levels. Although TSHI and TT4RI demonstrated statistically significant associations, their discriminatory performance was modest and should be interpreted with caution. These findings suggest that thyroid hormone sensitivity indices may complement conventional metabolic markers in the cross-sectional assessment of metabolic syndrome. However, prospective multicentre and mechanistic studies are needed to validate these associations and determine their clinical utility.

## Appendices

**Table 8:** . Supplementary Data (Variance Inflation Factor Assessment) Variance inflation factor analysis demonstrated no evidence of problematic multicollinearity among the variables included in the adjusted logistic regression models. VIF values ranged from 1.08 to 1.34, with the highest value observed for BMI (VIF=1.34), indicating minimal collinearity among the predictors.

Variable	VIF
Age	1.21
Sex	1.08
BMI	1.34
Smoking status	1.15
Physical activity	1.19
Model-specific thyroid sensitivity index	1.27
